# Supplementary material for: Ampk phosphorylation of Ulk1 is required for targeting of mitochondria to lysosomes in exercise-induced mitophagy
Source: Nat Commun. 2017 Sep 15;8:548. doi: 10.1038/s41467-017-00520-9 (PMC5601463; doi:10.1038/s41467-017-00520-9)
Supplement: Supplementary file 1 — Supplementary Information Supplementary figures [file 41467_2017_520_MOESM1_ESM.pdf]

File name: Supplementary Information
Description: Supplementary figures.

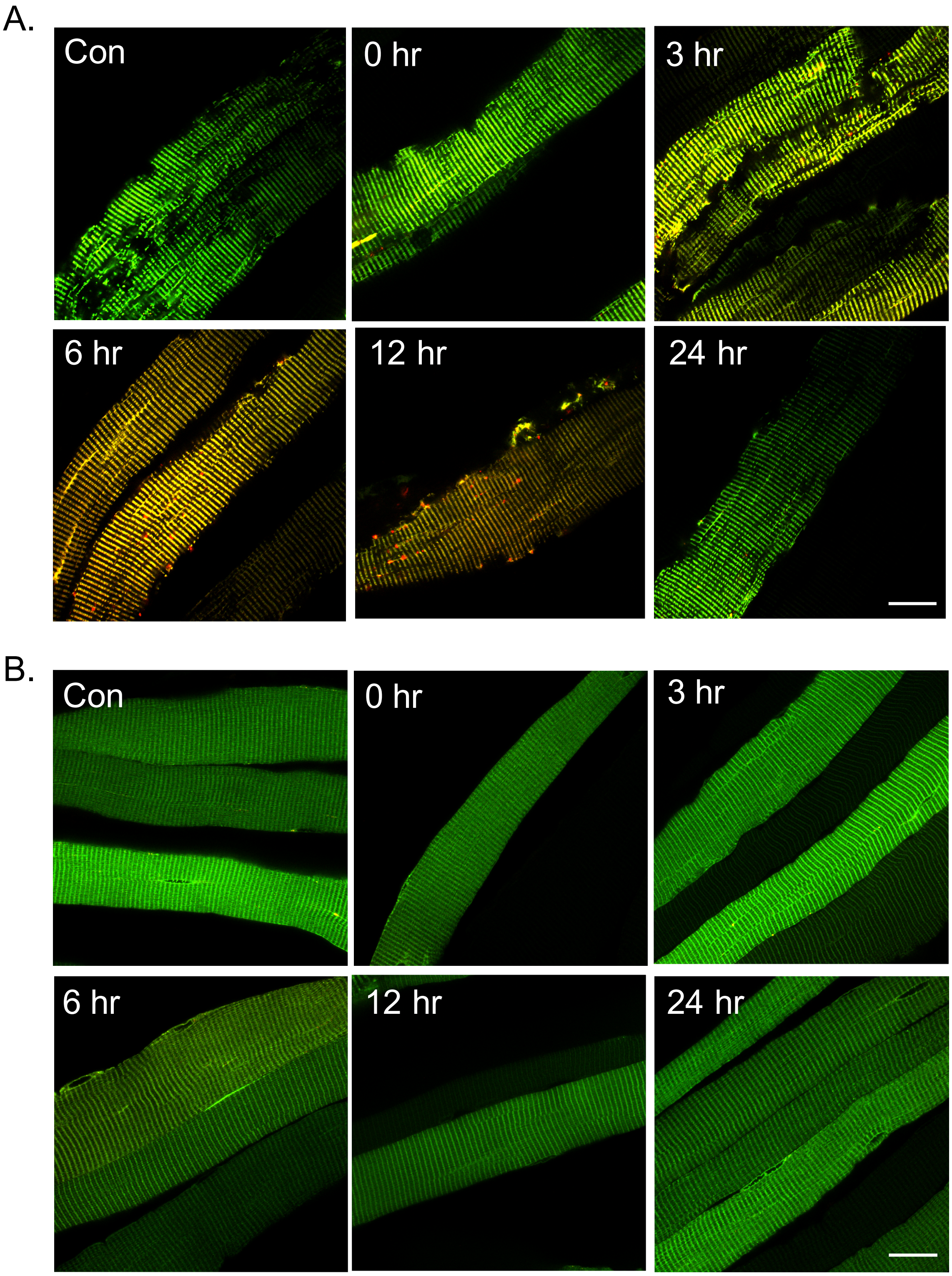

Supplementary Figure 1. Representative time-course images of mitochondrial oxidative stress following exercise. (A) Representative images of C57BL/6J (10-12 weeks) mouse FDB fibers transfected with *pMitoTimer* at time points following acute exercise. (B) Representative images of contralateral mouse FDB fibers transfected with *pER-Timer* at time points following acute exercise. Images are merged red and green channels. n=5-6 mice per time point. Scale bar = 20 μm.

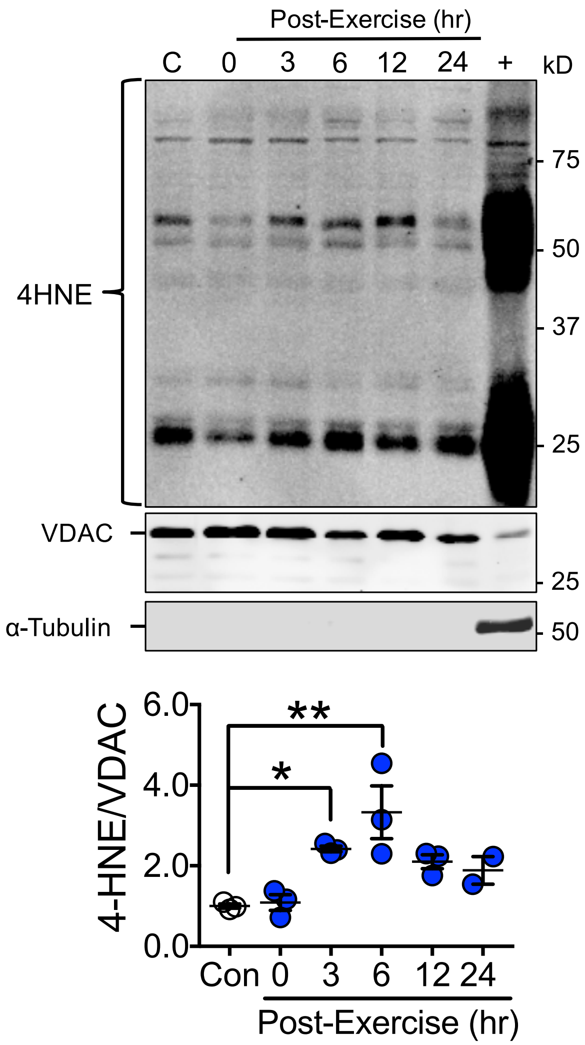

Supplementary Figure 2. Mitochondrial oxidative stress occurs during recovery from acute exercise. (A) Representative western blot of 4HNE of mitochondria-enriched lysates from WT gastrocnemius muscle in a time course following acute treadmill running in C57BL/6J mice (10-12 weeks). Detection of the mitochondrial specific protein Vdac and absence of the cytosolic protein α-Tubulin is indicative of pure mitochondria fractions. Positive control lane denoted by +, is whole kidney lysate 24 h following LPS injection. See also Fig. S5. (B) Quantification of whole membrane 4HNE abundance. Data presented as mean ± standard error of the mean. n=3 (except for 24 h, n=2). Results of One-way ANOVA is * $p < 0.05$, ** $p < 0.01$, F=7.67, DF=5.

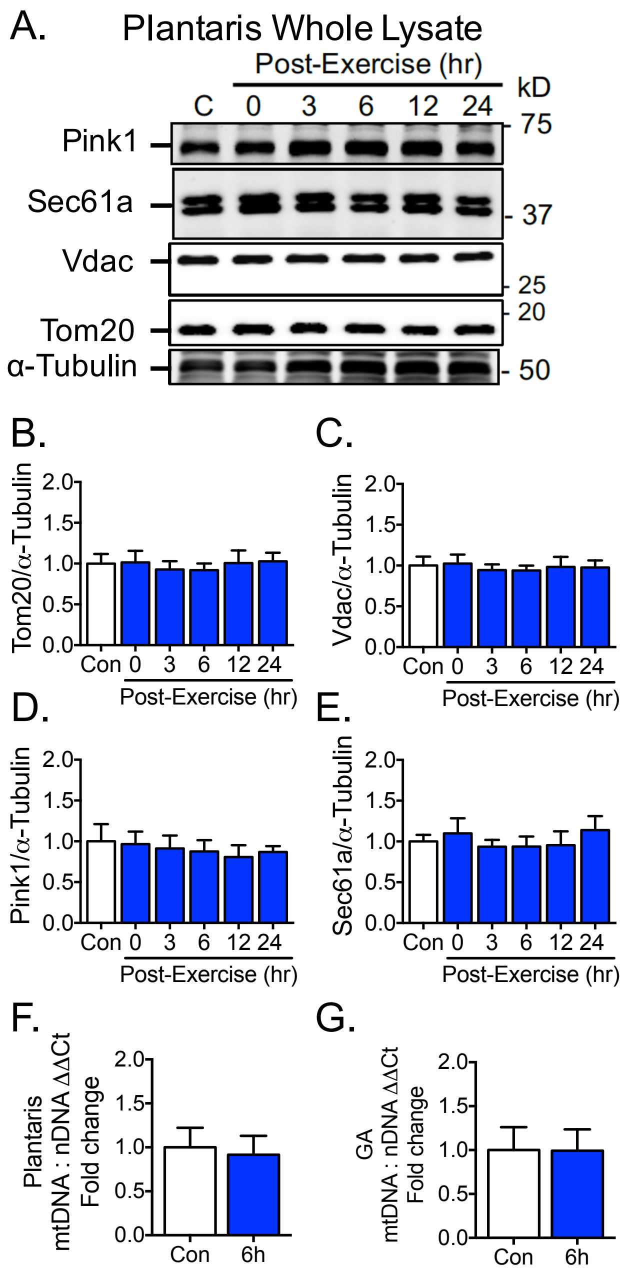

Supplementary Figure 3. Western blot for Pink1 and real-time PCR for mtDNA do not detect exercise-induced mitophagy in mouse muscle. (A) Representative western blot of the mitochondrial quality control protein, Pink1, and ER-content marker, Sec61a, and mitochondrial content markers, Vdac and Tom20, in WT mouse whole plantaris muscle lysate in a time course following acute treadmill running in C57BL/6J mice (10-12 weeks). See also Fig. S11. Quantification Tom20 (B), Vdac (C), Pink1 (D), and Sec61a (E) total protein abundance is expressed relative to α-Tubulin. Genomic DNA was isolated from GA and Plantaris muscles 6hr post exercise and the mtDNA marker *CytB* was compared to the nDNA marker *Gapdh* and no difference was seen compared to Con (F,G). Data presented as mean ± standard error of the mean. n=5 (except for 24 h, n=4), results of One-way ANOVAs are F=0.15 (B), F=0.12 (C), F=0.2 (D). F=0.37 (E), F=1.08 (F). and F=1.15 (G). DF=5.

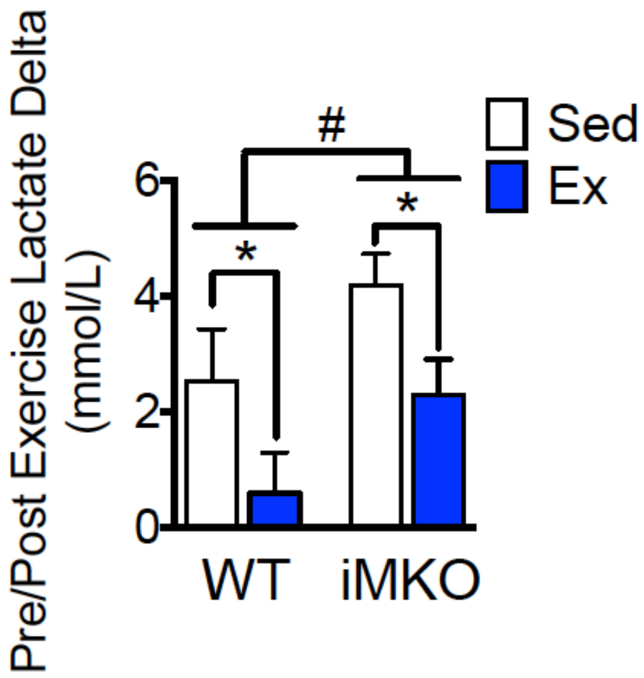

Supplementary Figure 4. Exercise capacity test pre vs. post lactate delta in iMKO. The delta between pre and post exercise capacity blood lactate is compared via two-way anova. Data presented as mean ± standard error of the mean. n=3 (WT) and n=4 (iMKO) (18-20 weeks). * $p < 0.05$ for sedentary vs. exercise comparisons. # $p < 0.05$ for between group comparisons.

Uncropped Western Blots of Acute Ex Timecourse from Fig. 1

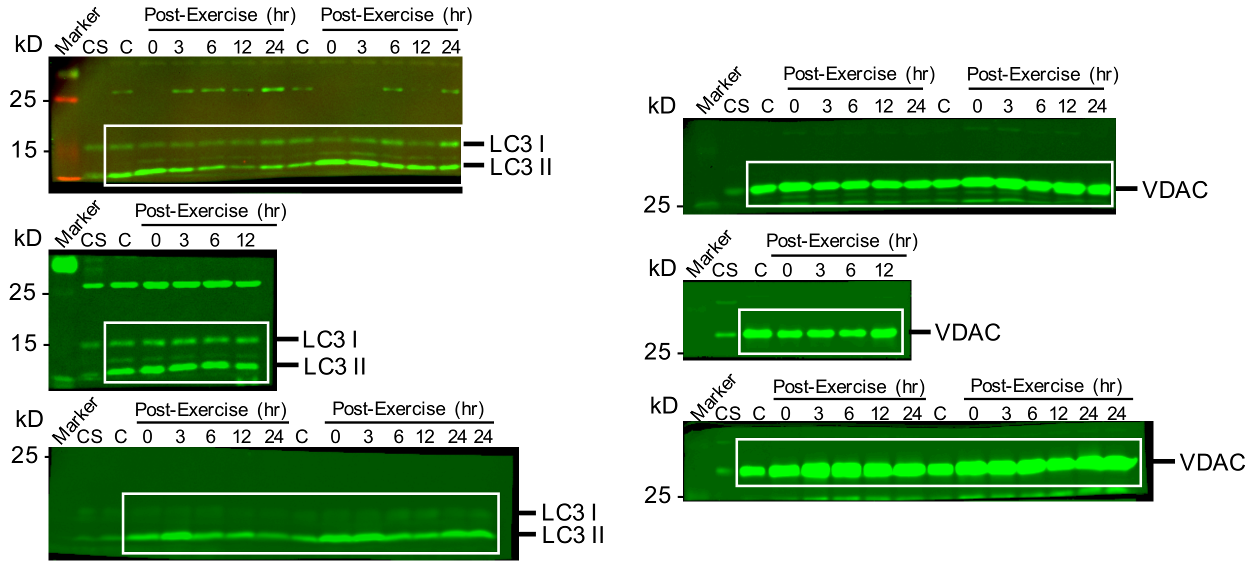

Uncropped Western Blots of Acute Ex Timecourse from Supplemntary Fig. 2

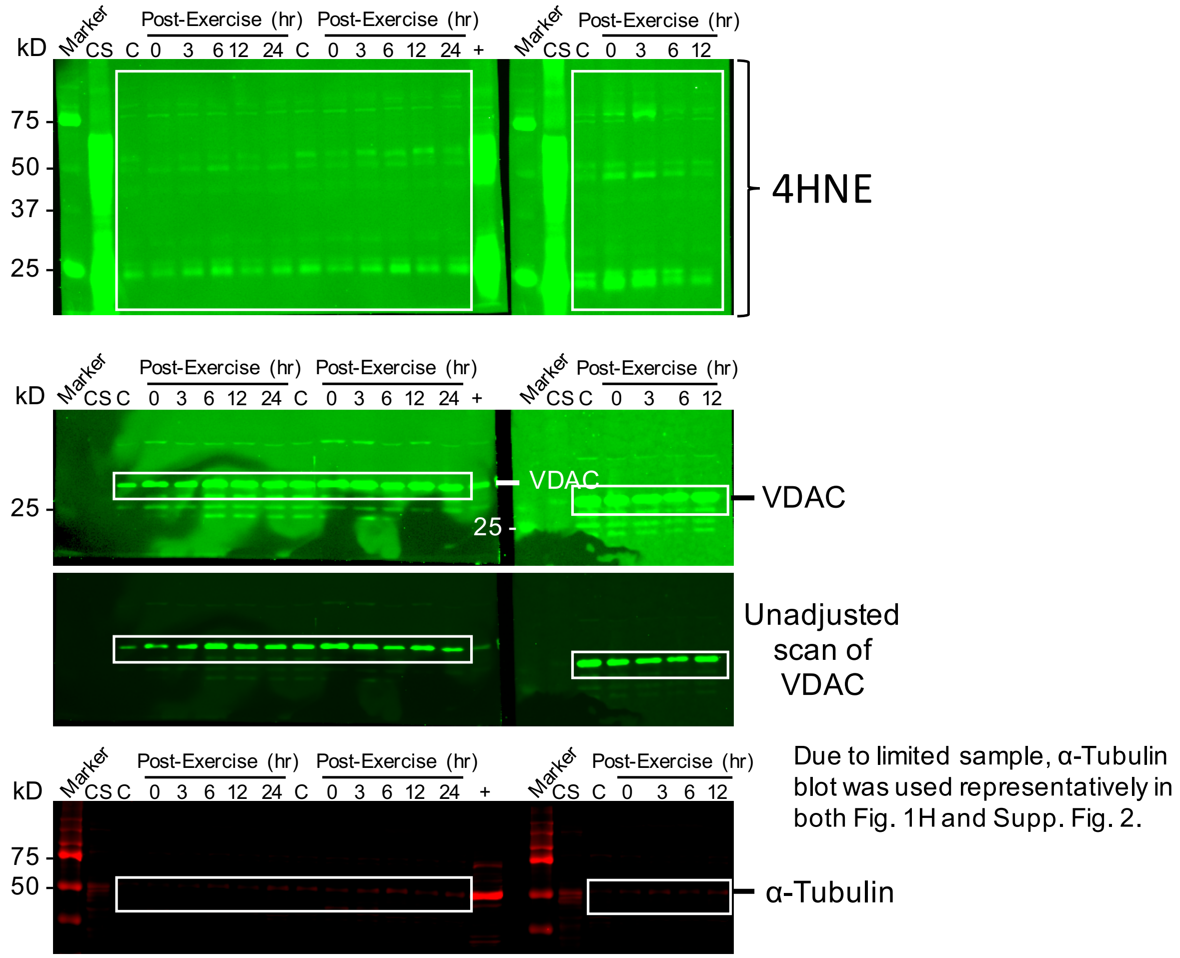

Supplementary Figure 5. Uncropped scans of western blots.

Uncropped Western Blots for Acute Exercise Timecourse from Fig. 2

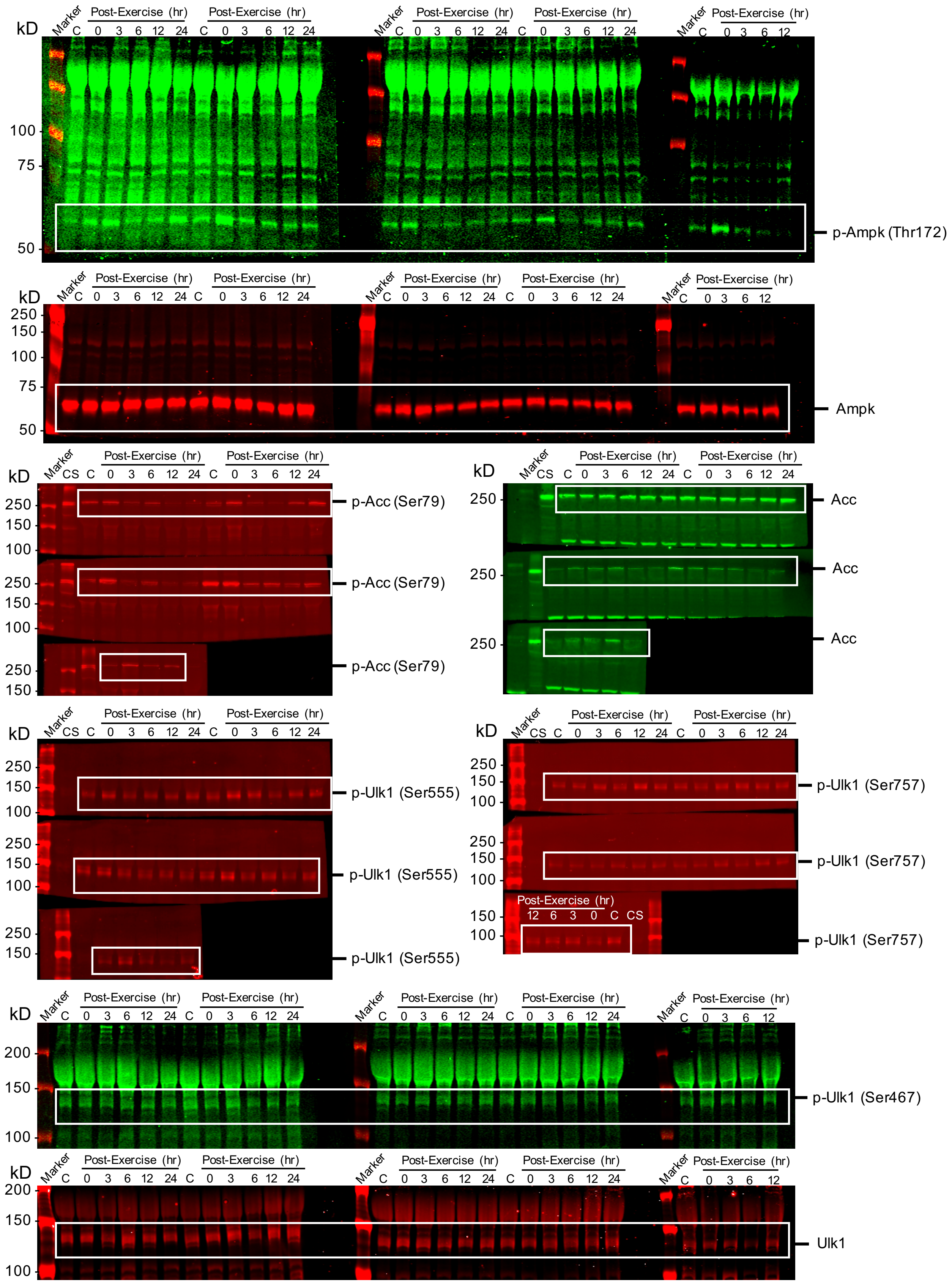

Supplementary Figure 6. Uncropped scans of western blots.

Uncropped Western Blots for Acute Exercise Timecourse from Fig. 2 continued

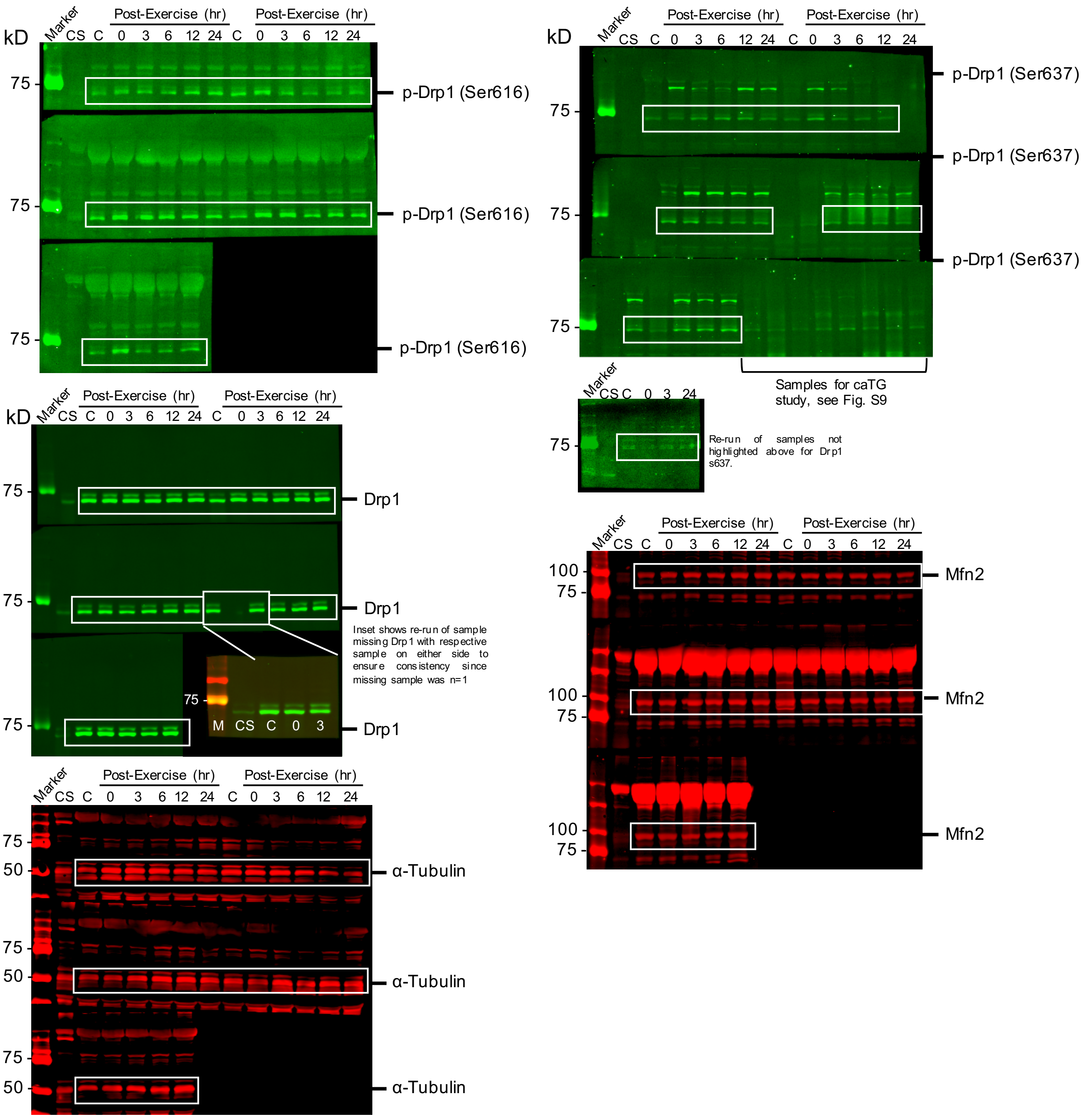

Supplementary Figure 7. Uncropped scans of western blots.

Uncropped Western Blots of dnTG from Fig. 3

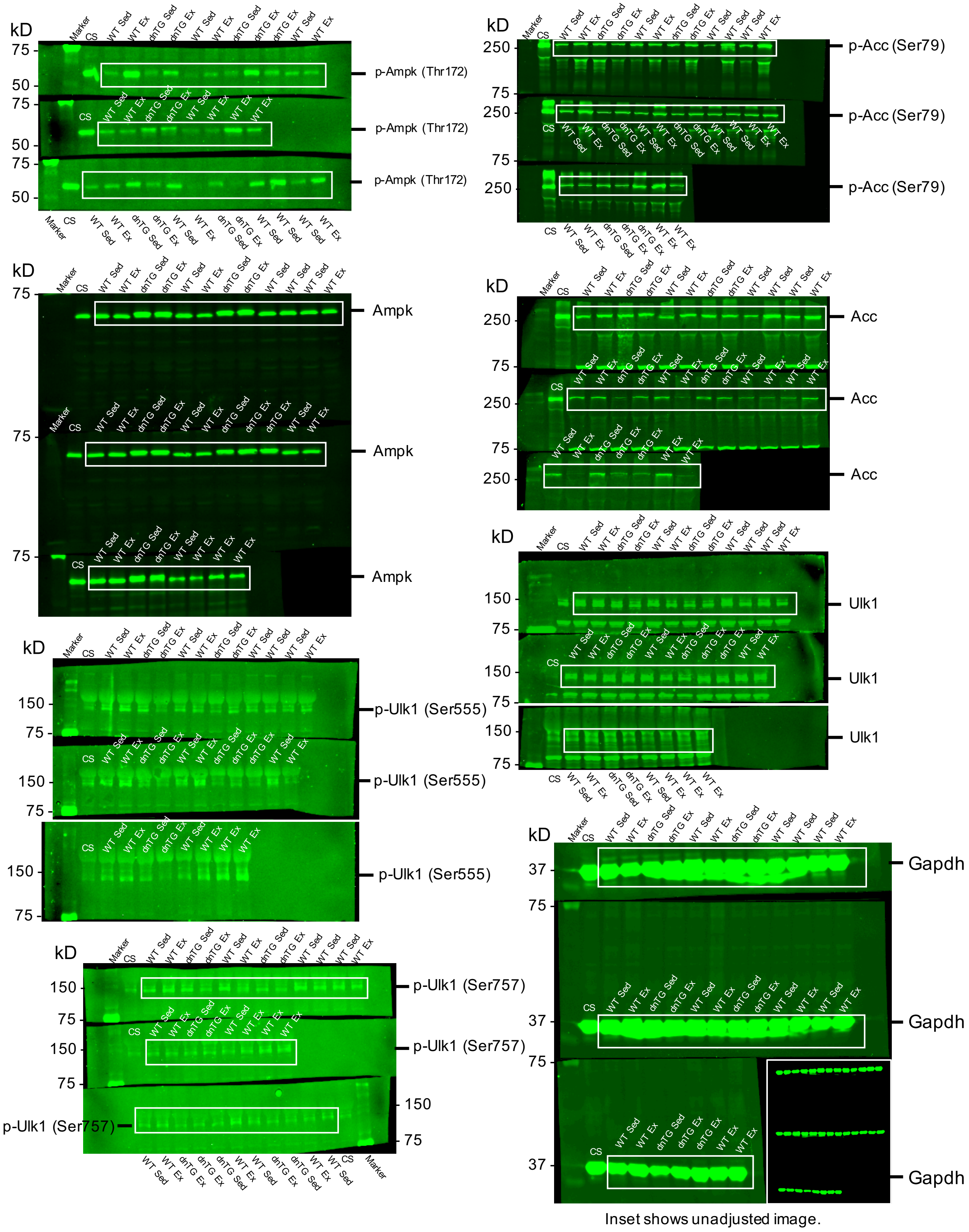

Supplementary Figure 8. Uncropped scans of western blots.

Uncropped Western Blots of caTG from Fig. 3

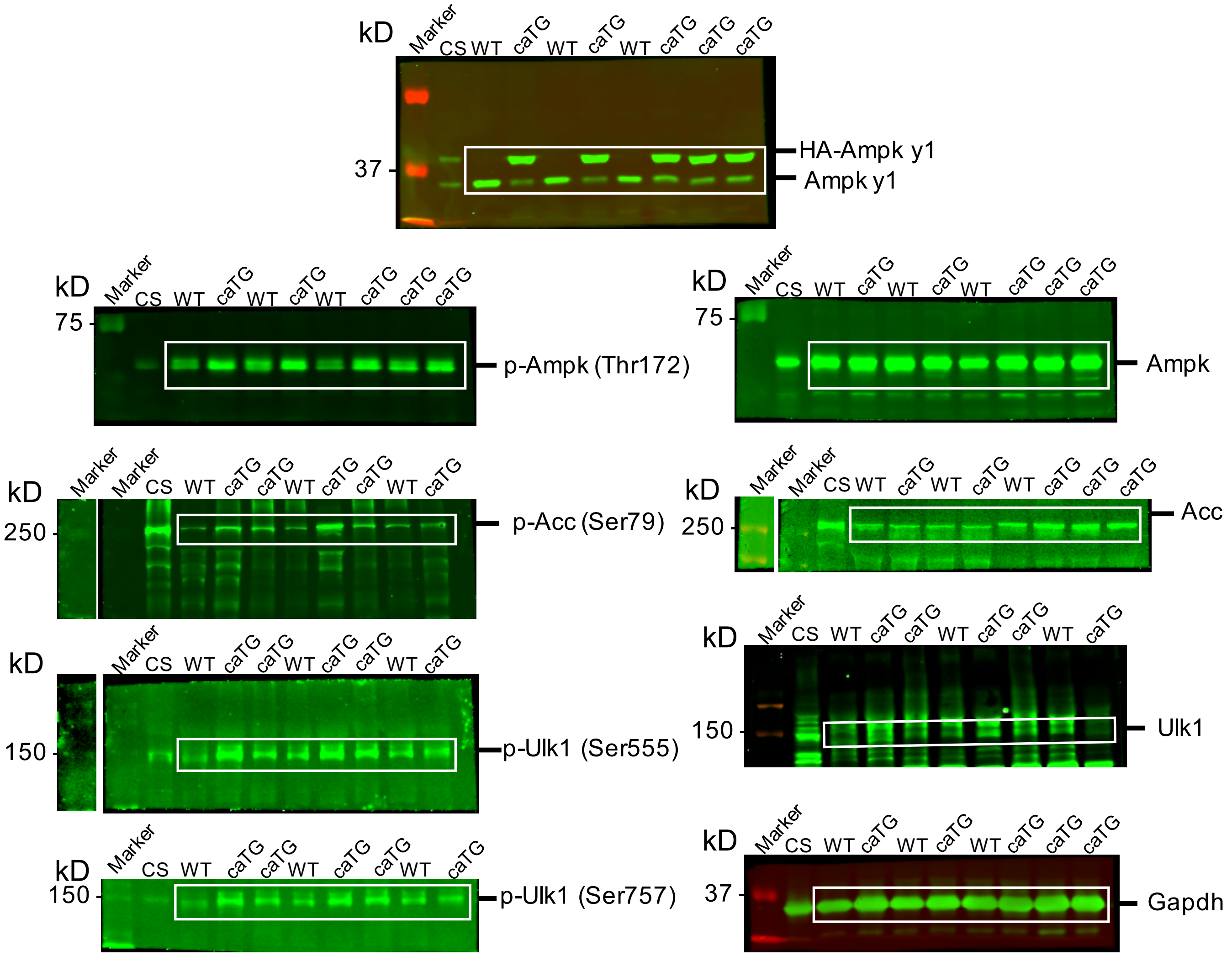

Supplementary Figure 9. Uncropped scans of western blots.

Uncropped Western Blots of dnTG from Fig. 4

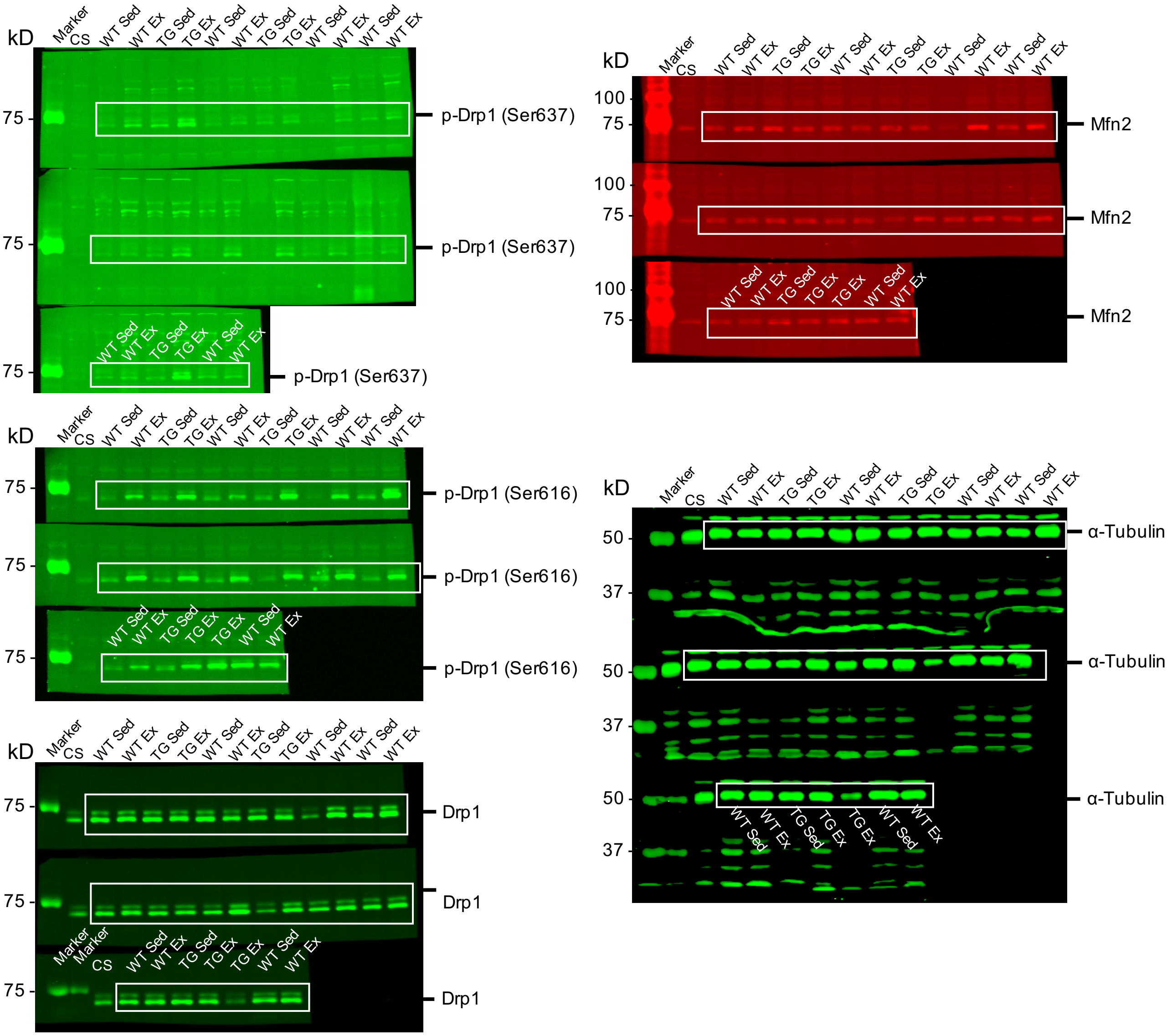

Uncropped Western Blots of caTG from Fig. 4

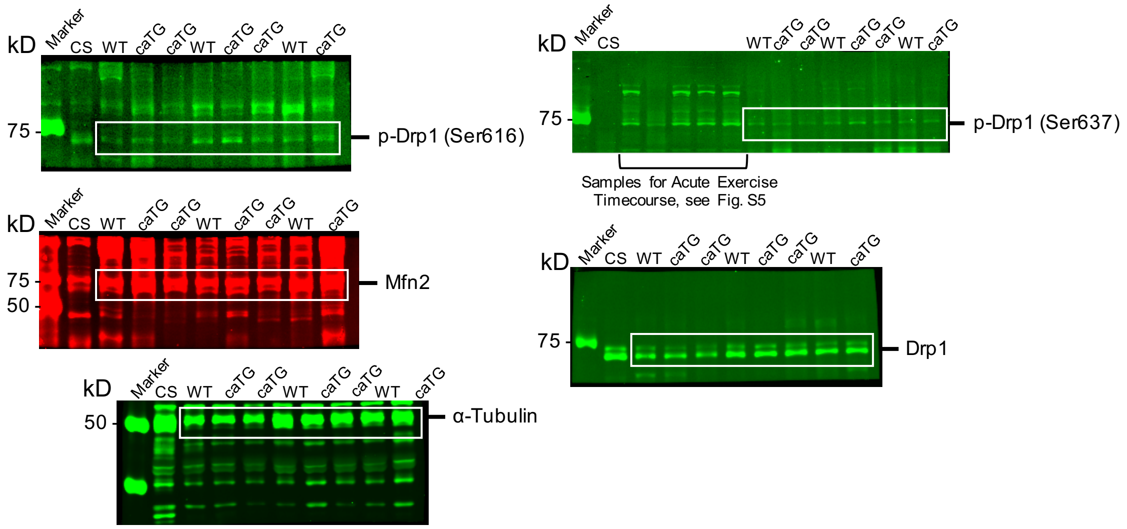

Supplementary Figure 10. Uncropped scans of western blots.

Uncropped Western Blots of Acute Ex Timecourse from Supplementary Fig. 3

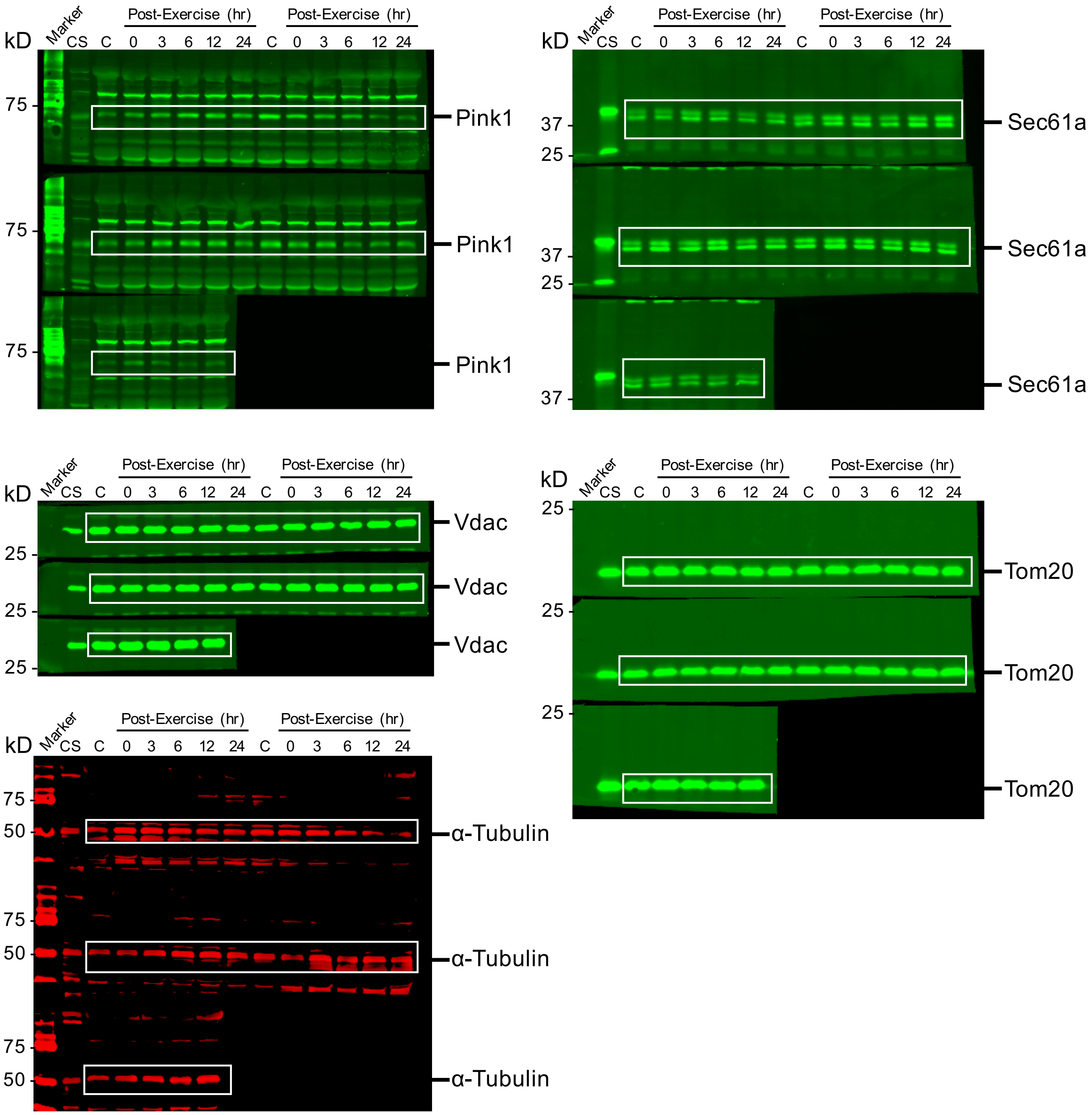

Supplementary Figure 11. Uncropped scans of western blots.
